# Effectiveness of one-on-one coaching in improving pressurized metered dose inhaler (pMDI) technique among COPD patients: a prospective clinical study

**DOI:** 10.1186/s12890-021-01627-y

**Published:** 2021-08-16

**Authors:** Jin Sun Kim, Nader Hashweh, Hannah Li, Salik Choudhary, Sadashiv Santosh, Edward Charbek

**Affiliations:** Division of Pulmonary, Critical Care and Sleep Medicine, Saint Louis University School of Medicine, 3635 Vista Ave, Saint Louis, MO 63110 USA

**Keywords:** COPD patients, Inhaler technique, One-on-one coaching, Educational intervention, pMDI

## Abstract

**Background:**

Incorrect use of inhalers among asthma and COPD patients is very prevalent. Yet, no single intervention is considered standard of care. We aimed to conduct a COPD-specific investigation of active one-on-one coaching as the educational intervention to improve pressurized metered dose inhaler (pMDI) technique and COPD symptoms management.

**Methods:**

COPD patients who have pMDI in their treatment regimen were enrolled in this prospective study using the Global Initiative for Chronic Obstructive Lung Disease criteria. After rapid cognitive screen, inhaler technique was assessed and an active one-on-one coaching was provided utilizing the 12-step American Thoracic Society instructions. Patients’ inhaler technique was assessed and scored again at their regular follow-up visits.

**Results:**

One hundred and one patients participated in the study. The percentage of pMDI misuse decreased from 43.5% pre-teaching to 12.9% post-teaching (binomial test *p* value < 0.001). The mean number of errors decreased from 3.1 errors pre-teaching to 1.7 errors post-teaching (paired t-test *p* value < 0.001). The number needed to treat was 3.3 patients to prevent one misuse. Patients with an impaired cognitive function were more likely to have inhaler misuse in general and less likely to improve their technique when provided training.

**Conclusions:**

This study reveals that many COPD patients have incorrect pMDI techniques that can be improved with a short training in the clinic.

*Trial Registration***:** Not applicable.

## Background

Chronic obstructive pulmonary disease (COPD) is a disease characterized by persistent respiratory symptoms due to airway abnormalities, leading to 3 million deaths a year [6]. Economically, COPD is calculated to cost the United States $32 billion in direct costs and an additional $20 billion in indirect costs. COPD also carries a high social burden, being the fifth leading cause of disability-adjusted life years lost globally and the second leading cause in the U.S. [6].

Alongside smoking cessation, pharmacotherapy is the mainstream treatment method for prevention of COPD exacerbations and reduction in loss of lung-function [3]. Most standard therapies, including beta-agonists, anti-muscarinics, and corticosteroids, may be administered via inhalation. Studies have shown that the inhalation route produces less adverse events compared to systemic dosing [3]. Hence, inhalers play a critical role in COPD management, serving as the principal medium for medication. These inhaled drugs are effective in providing symptomatic relief, improving quality of life, and reducing exacerbations [10, 12].

The correct use of inhalers is vital to achieve efficacy in treatment of COPD. Correct inhaler technique enables medications to properly reach the lungs and manifest its effects. Confidence of COPD patients in their inhaler technique has shown to be directly related to their treatment adherence and health status [1]. Unfortunately, the literature reports a high prevalence of poor inhaler technique in COPD and asthma patients [5, 8, 18, 23]. A recent study demonstrated that correcting poor inhaler technique improves the quality of life of COPD patients, as evidenced by improved FEV1 and COPD assessment test (CAT) scores [11]. Other comprehensive education programs have shown similar results [24]. This highlights the importance of precise inhaler technique for improving overall COPD management.

The aim of this study is to assess the effect of one-on-one coaching in improving the pMDI technique of COPD patients. Currently, there is no educational method that is standard of care. This study strives to show that a simple intervention—a brief pMDI technique training—can be a quick and low-cost solution to improving inhaler technique amongst COPD patients.

## Methods

This study was a prospective clinical study on COPD patients from an outpatient pulmonary clinic at Saint Louis University Hospital, assessing pMDI technique at baseline and after a short in-office education program. Patients were enrolled from June 2018 to April 2019 during their regular clinic visit. Inclusion criteria were adults aged ≥ 18 years and ≤ 90 years with a diagnosis of COPD who were regularly using pMDI with or without spacers. The Global Initiative for Chronic Obstructive Lung Disease (GOLD) criteria for airflow limitation (FEV1/FVC < 0.70) was utilized for diagnosis of COPD using patients’ most recent PFTs (Singh et al., 2019). Exclusion criteria were treatment of COPD exclusively without use of pMDIs; diagnosis of COPD but not meeting GOLD criteria; concurrent diagnosis of asthma; age < 18 years or > 90 years. Written informed consent was obtained from patients or surrogates. The study was approved by the Institutional Review Board (IRB approval number: 29266).

After enrollment, all patients underwent a rapid cognitive screen (RCS), a screening tool for cognitive decline, during their initial visit. Each patient’s pMDI technique was assessed utilizing the American Thoracic Society (ATS) recommended steps on using metered dose inhalers (MDIs) (Table 1). In evaluating the participant’s technique, each step was graded as correct or error. Following ATS guidelines, patients were labeled as having misuse of pMDIs if they had less than 75% (less than 9 out of 12) of the steps correct. All patients were then provided in-person, one-on-one coaching on how to correctly use the inhaler.

**Table 1:** ATS guidelines on correct MDI use

1	Put the metal canister into the “boot” making certain it is seated correctly
2	Shake the inhaler several times. This mixes the propellant and medicine
3	Remove the cap off from the mouthpiece
4	Breathe out to the end of a normal breath
5	Hold the inhaler in its upright position (with the mouthpiece at the bottom)
6	Put the mouthpiece in your mouth, past your teeth and above your tongue. Close your lips around the mouthpiece so that the medication does not go in your eyes
7	While breathing in slowly and deeply through your mouth, fully press down once on the top of the metal canister of your inhaler
8	Hold your breath for 5 to 10 s
9	Breathe out slowly
10	If you take more than one spray, wait 15 to 30 s (or as directed in the package insert) before taking the next puff. Then repeat steps 3–9
11	Replace the cap on the mouthpiece after you are finished
12	If you are inhaling a steroid, rinse your mouth out with water, swish, gargle and spit

Three of our research members were trained by a by a Pulmonary & Critical Care physician to provide one-on-one coaching on how to correctly use the pMDI. All teachers followed the ATS guidelines and used the exact wording from Table 1. A physical demonstration was provided at the same time with a sample empty inhaler. The coaching took about 5 min per patient. After coaching, each patient was asked to repeat his or her understanding of the coaching. If any steps were mistaken or omitted, the patient was instructed until he or she could repeat the correct steps.

On the next follow-up clinic visit, pMDI technique was evaluated again using the same scoring system. There was no specified duration for follow-up, and each patient was seen during his or her next regular clinic visit.

The primary outcome of change in pMDI technique with coaching was evaluated using two metrics: (1) change in misuse (i.e. the frequency of transition from misuse to correct use vs from correct use to misuse), and (2) change in the number of errors inhaler technique (as defined above). The secondary outcome was change in CAT score, which measures subjective COPD symptoms, and change in pulmonary function test (PFT) measurements before and after correct inhaler technique training if applicable.

CAT scores were measured routinely at each office visit, during each patient’s initial and follow-up visit. PFTs were not measured on the same day of patients’ initial or follow-up visits, but were measured following each patient’s unique clinical timeline. Patients typically had PFTs done once a year when they were guided to do so by their pulmonologists. Since our study only followed patients up to a year after their initial visit, we could not compare pre- and post-study PFT results for all patients.

Change in misuse was evaluated using the binomial test for paired nominal data (an exact alternative of McNemar’s test to account for small sample sizes). Change in the number of errors was evaluated using a paired t-test. To account for patients lost to follow-up, sensitivity analyses were performed. All patients with misuse at baseline who did not follow up were assumed to have continued misuse, and those with correct use were assumed to have continued correct use. To account for lack of control group, the possibility of spontaneous improvement in inhaler technique over time was assessed using linear and logistic regressions as described in Results. All statistical analysis was done using Stata/IC, release 14 (StataCorp, College Station, Texas).

## Results

One hundred one patients met inclusion and exclusion criteria, and were enrolled in the study. Of the 101 patients enrolled, 62 (61%) completed their follow-up evaluation and were included in the analysis of the primary outcome. Baseline characteristics (Table 2) for those with followup were similar to the entire enrolled cohort.

**Table 2:** Baseline characteristics

	Entire sample (n = 101)	Patients with followup (n = 62)
Age (years)	63.77 ± 7.17	64.24 ± 7.64
Number of inhalers	2.59 ± 0.59	2.52 ± 0.57
FEV1 (L)	1.29 ± 0.56 (n = 94)	1.28 ± 0.53 (n = 18)
FEV1% expected	50.85 ± 19.24 (n = 96)	51.83 ± 18.77 (n = 18)
CAT score	23.09 ± 7.84 (n = 95)	23.71 ± 7.45 (n = 59)
Number of errors	3.17 ± 1.57	3.11 ± 1.54
Years of inhaler use	8.67 ± 10.41	8.95 ± 10.8
Follow-up interval (months)	NA	4.69 ± 2.6
FEV1/ FVC	49.12 ± 12.52 (n = 81)	
Male sex	40 (40%)	23 (37%)
Active smoking	34 (34%)	19 (31%)
LABA	81 (80%)	51 (82%)
LAMA	84 (83%)	51 (82%)
ICS	68 (67%)	40 (65%)
SABA	92 (91%)	57 (92%)
SAMA	6 (6%)	4 (6%)
Montelukast	2 (2%)	2 (3%)
Theophylline	1 (1%)	0 (0%)
*Race*		
Asian	3 (3%)	0 (0%)
Black	55 (54%)	34 (55%)
White	38 (38%)	26 (42%)
Other	5 (5%)	2 (3%)
*RCS Score*	Entire sample (n = 62)	–
Normal	28 (45%)	–
Mild Impairment	19 (31%)	–
Dementia	13 (21%)	–
Unknown	2 (3%)	–

*FEV1* forced expiratory volume in one second, *CAT score* COPD assessment test score, *LABA* long-acting beta agonist

In the primary analysis (Table 3, row 1), the percentage of misuse from the initial visit was 43.5%. Some of the most commonly omitted/mistaken steps were steps 4 and 7, which are both crucial steps to drug delivery. Meanwhile, steps 3 and 9 were less likely to be omitted. The percentage of misuse post-teaching decreased to 12.9% (binomial test *p* value < 0.001). The mean number of errors decreased from 3.1 errors pre-teaching to 1.7 errors post-teaching (paired t-test *p* value < 0.001). The number needed to treat was 3.3 patients to prevent one misuse.

**Table 3:** Primary analysis and sensitivity analysis of misuse

	Pre-teaching	Post-teaching	Pre-to-post change (95%CI)	*p* value	Number needed to treat
Misuse (primary analysis)	27 (43.5%)	8 (12.9%)	− 30.6% (− 17.6% to − 43.7%)	< 0.001	3.3
Misuse (sensitivity analysis)	46 (45.5%)	27 (26.7%)	− 18.8% (− 10.2% to − 27.4%)	< 0.001	5.3
Errors	3.1 ± 1.5	1.7 ± 1.4	− 1.4 (− 1.8 to − 1)	< 0.001	

Misuse is reported as number (%). Errors is reported as mean ± SD

Due to the high rate of loss to follow-up, a sensitivity analysis was performed; all patients with misuse at baseline who did not follow up were assumed to have continued misuse, and those with correct use were assumed to have continued correct use. In the sensitivity analysis (Table 3, row 2), the frequency of misuse decreased from 45.5% to 26.7% (*p* value < 0.001). The number needed to treat increased to 5.3 patients to prevent one misuse. When compared to the cohort with followup, the frequency of misuse and mean number of errors at baseline were identical in the entire included sample.

Due to the lack of a control group, the possibility of spontaneous improvement over time was assessed by examining if years of prior inhaler use predicted baseline misuse or baseline number of errors. Over a 5-year horizon, years of inhaler use had essentially nil relationship with frequency of misuse (odds-ratio for misuse = 0.9 per year of inhaler use, *p* value 0.6) and with number of errors (change in number of errors = − 0.05 errors per year of inhaler use, *p* value = 0.85). Since misuse and number of errors seem to undergo very little spontaneous change over a period of years, it is extremely unlikely that the dramatic changes seen in this study with a mean follow-up of 4.7 months could have occurred in the absence of the intervention.

The interval length between patients’ initial and follow-up visit correlated with improvement on pMDI technique. Patients were followed up to 12 months from their initial visit, and this was analyzed in 3-month intervals (Table 4). The percentage of patients with improvement in inhaler use decreased as the follow-up interval increased. This trend was not seen in the 9–12 month follow-up category, however this was difficult to assess since there were only 4 patients in this bracket (Table 4).

**Table 4:** Follow-up interval and improvement of pMDI technique

Follow-up interval	Number of patients	Number of patients with improvement at follow-up	Percentage (%) of patients with improvement
1–3 months	25	9	36%
3–6 months	25	6	24%
6–9 months	18	2	11%
9–12 months	4	2	50%

**Fig. 1:**
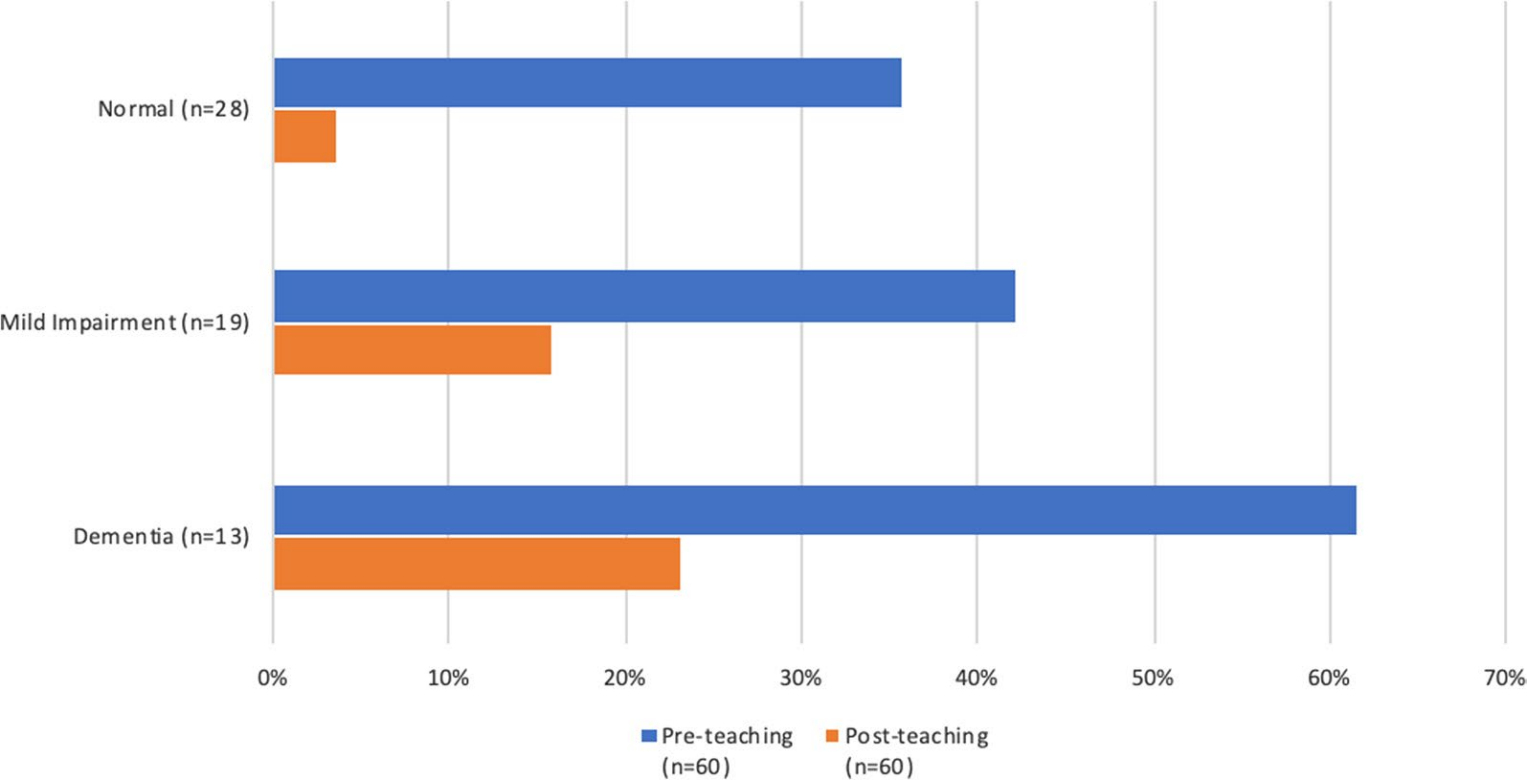
Percent patients with pMDI misuse by RCS score

The impact of cognitive status on improvement of pMDI technique with teaching was studied using baseline RCS scores of patients with baseline misuse and follow-up data (Table 2). The frequency of pMDI misuse at baseline was numerically higher in patients with mild cognitive impairment (42% misuse) and dementia (62% misuse) than those with normal cognition (36% misuse), although this difference was not statistically significant (*p* value = 0.3 by Chi-squared test) (Fig. 1). Of the 26 patients with baseline misuse and follow-up data, the fraction of patients whose pMDI technique improved to correct use (≥ 9 correct steps) was lower in those with mild cognitive impairment (63% improved) and dementia (63% improved) than those with normal cognition (90% improved), although this trend did not reach statistical significance (*p* value = 0.3 by Fisher’s exact test) (Fig. 1). Even amongst those with cognitive impairment, the majority of patients improved with teaching.

The impact of correct pMDI technique on COPD symptoms was examined using CAT scores (Table 2). Baseline number of errors (*p* value = 0.7 by linear regression) and baseline misuse (*p* value = 0.8 by t-test) did not predict baseline CAT score. In patients with baseline and follow-up CAT scores recorded, the overall score changed very little (mean change = − 0.4 ± 1.0 [SE], *p* value = 0.7). Amongst those with baseline misuse, those whose pMDI technique improved had similar changes in CAT score to those with continued misuse (+ 1.9 vs − 3.4, *p* value = 0.2).

There were not enough patients with PFTs before and after intervention for a meaningful analysis of the impact of teaching on spirometry.

## Discussion

This study demonstrates that in patients with COPD being treated with pMDIs, a simple 5 min intervention dramatically reduces pMDI misuse. Patients with an impaired cognitive function were more likely to have inhaler misuse in general and less likely to improve their technique when provided training. However, despite the lesser benefit observed in those with cognitive impairment, the difference was not statistically significant, and patients with mild cognitive impairment and dementia still appeared to benefit meaningfully. This corroborates findings in a study that found that an 8-day inhaler training program in the geriatric population led to an improved technique and CAT scores regardless of cognitive abilities [15].

The study participants were predominantly Black or White and had a wide range of therapeutic regimens, baseline CAT scores, and cognitive function (see Table 2). As a result, the results can be generalized to a broad COPD population. One limitation was the lack of data collected on comorbidities of the study participants; more baseline data would have improved our confidence in generalizability and repeatability. We highly recommend clinicians to provide reminder trainings for COPD patients who are coming in for regular clinic visits. This low cost intervention greatly benefits patients’ inhaler technique and hence COPD management. Reminder trainings are important because studies have shown that inhaler technique has not changed over the past several decades, remaining poor [22]. Regular evaluation of technique has been specifically recommended for the geriatric population to revisit the choice of inhalers and their limitations [4].

Despite the benefit in enhancing patients’ pMDI technique, the study found no difference in the changes in CAT scores between patients who had improved pMDI technique and those who did not. This might be attributed to the relative short-term duration of the study and the one-time follow-up assessment design of the study. Currently the literature on the effect of inhaler training on health outcomes is inconclusive: some studies have shown no association, potentially due to the short duration of these studies [16,17], while others suggested improved outcomes (decreased cough, decreased breathlessness upon exertion) [7, 8].

The main strength of this study is that it is a longitudinal, prospective study with patient evaluations performed before and after the intervention. In addition, the simplicity, ease, and low cost of the intervention should facilitate its application in routine patient care. This study also explores the efficacy of pMDI coaching among participants of different levels of cognition, an area that has not been well-researched in the literature.

The primary limitation of this study is its non-randomized design. Due to lack of a placebo group, the possibility that spontaneous improvement in the primary outcome could occur in absence of the intervention cannot be completely ruled out. However, it is shown that such spontaneous improvement in inhaler technique is unlikely to occur in such a short follow-up interval [7].

Another limitation is that about one third of patients were lost to follow-up. The most common reason for this was that many patients did not show up to their follow-up appointments or rescheduled their visit past our 12-month follow-up period. This was expected given the majority low-income patient population in urban St. Louis. Our study clinic also did not require a penalty for no-shows. Other reasons included patients relocating or changing insurances. However, patients lost to follow-up did not differ in baseline characteristics from those included in the primary analysis. Furthermore, results from the sensitivity analysis assuming zero improvement in those lost to follow-up were consistent with the primary results and confirmed the large effect size of the intervention.

Finally, this study was limited to pMDIs and did not investigate the technique of DPIs or SMIs. This was due to the wide usage of pMDIs—all COPD patients in the study clinic had at least one pMDI in their inhaler treatment regimen. DPIs and SMIs were purposefully left out for the benefit of having a stronger statistical analysis by simplifying the study. We recommend further studies to expand the investigation to include DPIs and SMIs to compare findings. A review of studies investigating DPI technique found misuse in participants, which confirms results from studies that did not differentiate based on inhaler type and this study that focused on pMDIs [14]. We also recommend reexamination of medical personnel’s knowledge of inhaler techniques as patients’ education is dependent on correct teaching [9].

The results of this trial should also be confirmed in future randomized studies. Such studies should include a control group and repeated assessments of inhaler technique over a longer follow-up period. Such studies would help determine the durability of the improvements seen with teaching and the impact of improved technique on respiratory symptoms and lung function over time. We also recommend collecting data on number of exacerbations and hospitalizations to investigate if improvement in inhaler technique affects these outcomes. One observational study has tied incorrect inhaler technique to more frequent exacerbations leading to hospitalization but has not investigated the effect of coaching on these outcomes [19]. There has also been studies that showed that in-person counseling sessions are significantly more effective and preferred than reading pamphlets or watching videos [2, 20, 21]. Hence, evaluating the efficacy and feasibility of pharmacists versus nurses versus providers versus other personnel administering the teaching would be another next step. Finally, further research is needed to determine whether alternative means of teaching, inhaler choices, or other interventions can be tailored to patients based on cognition function considering the high comorbidity of COPD and neurocognitive disorders [13].

## Conclusions

In conclusion, this study reveals that many COPD patients have incorrect pMDI techniques that can be improved with a short training in the clinic, including in patients with neurocognitive disorders. We recommend wide implementation of this one-on-one coaching in patients’ clinic visits.

## Data Availability

The datasets used and/or analyzed during the current study are available from the corresponding author on reasonable request.

## Abbreviations

COPD: Chronic obstructive pulmonary disease
MDI: Metered dose inhaler
pMDI: Pressurized metered dose inhaler
DPI: Dry powder inhaler
SMI: Soft mist inhaler
GOLD: Global initiative for chronic obstructive lung disease
ATS: American Thoracic Society
CAT: COPD assessment test
RCS: Rapid cognitive screen
PFT: Pulmonary function test
FEV1: Forced expiratory volume in one second
FVC: Forced vital capacity
LABA: Long-acting beta agonist
LAMA: Long-acting muscarinic antagonists
ICS: Inhaled corticosteroids
SABA: Short-acting beta agonists
SAMA: Short-acting muscarinic antagonists
